# Dynamics of triacylglycerol and EPA production in *Phaeodactylum tricornutum* under nitrogen starvation at different light intensities

**DOI:** 10.1371/journal.pone.0175630

**Published:** 2017-04-12

**Authors:** Ilse M. Remmers, Dirk E. Martens, René H. Wijffels, Packo P. Lamers

**Affiliations:** 1 Bioprocess Engineering & AlgaePARC, Wageningen University and Research, Wageningen, The Netherlands; 2 Biosciences and Aquaculture, Nord University, Bodø, Norway; Stazione Zoologica Anton Dohrn, ITALY

## Abstract

Lipid production in microalgae is highly dependent on the applied light intensity. However, for the EPA producing model-diatom *Phaeodactylum tricornutum*, clear consensus on the impact of incident light intensity on lipid productivity is still lacking. This study quantifies the impact of different incident light intensities on the biomass, TAG and EPA yield on light in nitrogen starved batch cultures of *P*. *tricornutum*. The maximum biomass concentration and maximum TAG and EPA contents were found to be independent of the applied light intensity. The lipid yield on light was reduced at elevated light intensities (>100 μmol m^-2^ s^-1^). The highest TAG yield on light (112 mg TAG mol_ph_^-1^) was found at the lowest light intensity tested (60 μmol m^-2^ s^-1^), which is still relatively low to values reported in literature for other algae. Furthermore, mass balance analysis showed that the EPA fraction in TAG may originate from photosynthetic membrane lipids.

## Introduction

Lipid production by phototrophic microalgae is only economically feasible today for high-value products, such as pigments and ω-3-fatty acids [1]. One of the major hurdles for commercial bulk microalgal lipid production is the low lipid yield on light [1–3].

Typically, microalgal lipid production is induced by nitrogen deficiency. When exposed to nitrogen deficient conditions, some microalgal species can accumulate triacylglycerol (TAG) up to 30–60% of dry weight [4–6]. However, nitrogen deficiency is often accompanied with impaired or even fully inactivated photosynthesis [4,7] and therefore results in a reduced TAG yield on light. For commercial production of TAGs, the TAG yield on light should be increased.

One of the most advertised microalgae for commercial lipid production is *Phaeodactylum tricornutum*. *P*. *tricornutum* is a salt-water diatom that is already cultivated at large scale for aquaculture. This microalgae produces large amounts of eicosapentaenoic acid (EPA; a long chain polyunsaturated fatty acid) and can produce TAG up to 30% of dry weight [4]. It is generally acknowledged, however, that high light exposure can severely affect the biomass and lipid productivity of this diatom [8–12]. However, clear consensus regarding the impact of light intensity on TAG and EPA productivity in *P*. *tricornutum* is still lacking. The aim of this work is to obtain insight in the relation between incident light intensity and the accumulation of TAG and EPA under nitrogen starvation.

## Material and methods

### Strain, medium and precultivation

*Phaeodactylum tricornutum* SAG1090-1b was obtained from the Culture Collection of Algae Goettingen University, Germany. Cultures were maintained in 250 mL shake flasks containing 100 mL filter sterilized (pore size 0.2 μm) medium. The medium was designed to reach a biomass concentration of 2.5 g L^-1^ and consisted out of 252 mM NaCl, 16.8 mM KNO_3_, 3.5 mM Na_2_SO_4_, 5 mM MgSO_4_ 7H_2_O, 2.4 mM CaCl_2_ 2H_2_O, 2.5 mM K_2_HPO_4_, 10 mM NaHCO_3_, 28 μM NaFeEDTA, 80 μM Na_2_EDTA 2H_2_O, 19 μM MnCl_2_ 4H_2_O, 4 μM ZnSO_4_ 7H_2_O, 1.2 μM CoCl_2_ 6H_2_O, 1.3 μM CuSO_4_ 5H_2_O, 0.1 μM Na_2_MoO_4_ 2H_2_O, 0.1 μM Biotin, 3.7 μM vitamin B1 and 0.1 μM vitamin B12. The medium contained 100 mM, 2-[4-(2-hydroxyethyl)piperazin-1-yl]ethanesulfonic acid (HEPES) as a pH buffer. Prior to cultivation, the pH was adjusted with KOH to pH 7.2.

Cultures were maintained in a cultivation chamber at 25°C, orbitally shaken at 120 rpm, illuminated at an incident light intensity of 40–60 μmol m^-2^ s^-1^ and a 16:8h light:dark (LD) cycle under enriched air (2% CO2). Three days prior to the start of the experiment, cultures were transferred to an orbital shaker incubator with an incident light intensity of 160 μmol m^-2^ s^-1^, 16:8h LD cycles and 2.5% CO2 enriched air.

### Photo bioreactor setup and operation

Experiments were performed in aseptic, flat-panel, airlift-loop reactors (Algaemist, Technical Development Studio, Wageningen University, the Netherlands). The reactor has a working volume of 400 mL, a light path of 14 mm and an illuminated area of 0.028 m^2^ (Schematic illustration of the reactor design is given in Breuer et al. [9]). Illumination was provided using 6 LED lamps with a warm white light spectrum, (BXRA W1200, Bridgelux, USA, emission spectrum given in the S1 Fig) under a 16:8h LD cycle. The incident photon flux density (PFD_in_) was measured with a LI-COR 190-SA 2π PAR (400–700 nm) quantum sensor (PAR, USA) and determined as the average over 28 points evenly distributed over the illuminated surface of the inside of the front glass panel of the reactor. The culture was continuously supplied with sterile moisturized air at 0.4 L min^-1^. pH was maintained at 7.2 by on-demand sparging of CO_2_ to the airflow and temperature was kept constant at 20°C. Reactor medium was similar to the preculture medium, with modifications to the amount of KNO_3_ (5 mM instead of 16.8 mM) and exclusion of HEPES. 5 mM of KNO_3_ is sufficient to reach a biomass concentration of 1.2 g L^-1^ (assuming a cellular nitrogen fraction of 10% per dry weight, measurements not shown). Per day, a maximum of 2 drops of 1% (v/v) antifoam (Antifoam B, J.T. Baker) were added manually to the reactor when foaming was visible.

Reactors were heat sterilized (60 min at 121°C) and inoculated at a biomass concentration of approximately 0.5 g L^-1^ of dry weight and grown at an incident light intensity of 100 μmol m^-2^ s^-1^. Once the biomass concentration reached 1 g L^-1^, which was typically three days after inoculation, the set points for incident light intensity were changed to the desired values (60, 100, 250, 500, or 750 μmol m^-2^ s^-1^ incident light intensity). The day at which the light intensity was changed to the experimental set point was regarded as start of the experiment, and therefore referred to as t = 0. Periodically, samples (approximately 20 mL culture volume) were taken aseptically and analysed for dry weight concentration, triacylglycerol (TAG) and total fatty acid (TFA) concentration and composition, dissolved (residual) nitrate Photosystem II (PSII) maximum quantum yield (F_v_/F_m_) ratio, and absorption cross section. After taking a sample, the culture volume was restored by adding 0.2 μm-filtered reactor medium to ensure that the pH probe remained submerged. Depending on whether the culture was in N-replete phase or N-depleted phase, this medium contained either 5 mM KNO_3_ or no nitrogen, respectively.

Five different incident light intensities were selected that cover both low light and light saturation (light saturation point for *P*. *tricornutum* was found at 150–200 μmol m^-2^ s^-1^ [13–15]). Three photo bioreactor experiments were conducted in biological duplicate to test reproducibility[16].

Measurements for dry weight, F_v_/F_m_ and dissolved nitrogen were performed in technical triplicates. Measurements for the light absorption spectrum, lipid content and composition were performed in technical duplicate.

### Biomass analysis

Samples were taken at regular time points to monitor biomass dry weight concentration gravimetrically as described by [17]. Light absorption spectrum was measured in a double-beam spectrophotometer (UV-2600, Shimadzu, Japan) equipped with an integrated sphere (ISR-2600). The average dry weight-specific optical cross section (α_C_ in m^2^ g^-1^) was calculated from the obtained absorption spectrum as described by [18]. The F_v_/F_m_ ratio was measured using an AquaPen-C AP-C 100 FluorPen fluorometer (Photon Systems Instruments, Czech Republic) equipped with an orange LED emitter (excitation light 620 nm) as described by [19].

The triacylglycerol (TAG), membrane lipid (ML) and total fatty acid (TFA) content and fatty acid composition were determined by lipid extraction (chloroform:methanol mixture), lipid class separation using a silica column, transesterification and subsequently quantified using gas chromatography (GC-FID) as described by Breuer et al. [9]. The dissolved nitrate concentration was measured with an AQ2 nutrient analyser (Seal Analytical, USA) as described by Benvenuti et al. [20].

### Calculating yield and productivity

The time averaged volumetric productivity (*r*_*i*_(*t*_0_ → *t*)) was calculated by dividing the amount of formed product (i: biomass, triacylglycerol, EPA) over cultivation time according to Eq 1;

$$r_{i}\left(t_{N=0}\to t\right)=\frac{C_{i}\left(t\right)-C_{i}\left(t_{N=0}\right)}{t-t_{N=0}}$$ (1)

The time averaged yield of TAG on light (*Y*_*TAG*,*ph*_(*t*_0_ → *t*)) on each time point was calculated by dividing the amount of TAG produced in that period by the amount of light supplied in that period [21]. In addition, the photon costs for initial biomass production until nitrogen depletion were incorporated by assuming that this biomass was made with a theoretical net photosynthetic yield, Y_x,ph_, of 1g mol_ph_;

$$Y_{TAG,ph}\left(t\right)=\frac{TAG\left(t\right)-TAG\left(t_{N=0}\right)}{\frac{I_{0}\cdot \left(t-t_{N=0}\right)}{z}+\frac{C_{x,N=0}}{Y_{x,ph}}}$$ (2)

In Eq 2, TAG(t) and TAG(t_N = 0_) represent the TAG concentration at time point t and the moment of nitrogen starvation, respectively. The exact time of nitrogen starvation (t_N = 0_) was estimated by interpolation of the biomass concentration to 1.2 g L^-1^. I_0_ is the incident light intensity, z is the reactor depth and C_x,N = 0_ is the biomass concentration at the onset of nitrogen starvation.

## Results and discussion

### Effect of light on biomass growth

**Fig 1:**
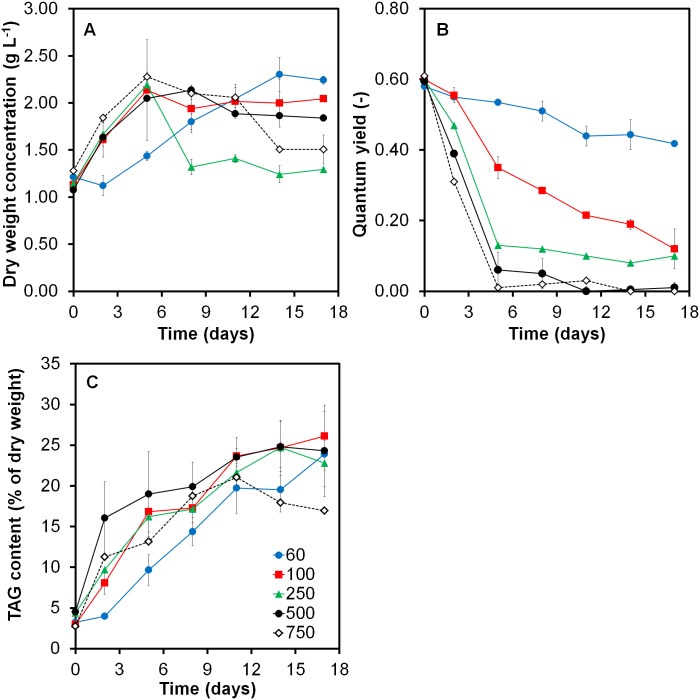
Impact of five different incident light intensities on (A) the biomass concentration, (B) F_v_/F_m_ ratio and (C) the TAG content over time. Light was supplied at intensities of 60 μmol m^-2^ s^-1^ (blue circles), 100 μmol m^-2^ s^-1^ (red squares), 250 μmol m^-2^ s^-1^ (green triangles), 500 μmol m^-2^ s^-1^ (black circles) and 750 μmol m^-2^ s^-1^ (open diamonds). Sufficient nitrogen was added to reach biomass concentrations of 1.2 g L^-1^, thereafter cells were exposed to nitrogen starvation. Average values of duplicate reactor runs are shown, except for the cultures exposed to 250 and 750 μmol m^-2^ s^-1^ (n = 1). Error bars indicate the standard deviation of both cultures from the average value.

The impact of five different light intensities on biomass, TAG and EPA productivity during nitrogen starvation was assessed in batch-wise operated flat-panel photo bioreactors that were subjected to 16:8h light:dark (LD) cycles. Results for dry weight, quantum yield and TAG content are shown in Fig 1.

Sufficient nitrogen was added to reach a biomass concentration of 1.2 g DW L^-1^. After that biomass concentration was reached, all nitrogen was used and the culture was exposed to nitrogen starvation. The biomass concentration further increased up to 2.2 g L^-1^ of dry weight, which was due to the accumulation of TAG (Fig 1) and carbohydrates (S1 Table). The maximum achieved biomass concentration was reached 5 days after nitrogen starvation started, except for the culture exposed to the lowest light conditions, which reached the maximum biomass 14 days after nitrogen starvation started. The maximum reached biomass concentration was 2.15±0.1 g L^-1^, independent of the supplied light intensity (Fig 1A). Other studies showed similar findings for the freshwater microalgae *Acutodesmus obliquus* under nitrogen starvation [4].

Cultures that are light limited are expected to show an increase in biomass productivity upon increasing light intensities [9]. However, our results show no differences in productivities for the cultures exposed to 100 μmol m^-2^ s^-1^ and higher (Fig 1). This similarity in productivity might be explained by photo inhibition that occurred from 100 μmol m^-2^ s^-1^ onwards. As soon as the photosynthetic machinery becomes inhibited, further increasing light intensity would not enhance the productivity anymore [22]. According to Fig 1B, a strong decline in F_v_/F_m_ was observed (from 0.6 to 0) for cultures exposed to light intensities above 100 μmol m^-2^ s^-1^ immediately after nitrogen starvation. A decline in F_v_/F_m_ is generally acknowledged with reduced photosynthetic efficiency or photo damage [19,23,24]. Geider et al. [13] presented a light curve for *P*. *tricornutum*. Similar to our findings, they reported that from 100 μmol m^-2^ s^-1^ onwards light saturation occurs in *P*. *tricornutum*. In addition, Acién Fernández et al. [25] performed outdoor growth experiments with *P*. *tricornutum*, where the maximum biomass productivity was reached at average irradiances of 50–150 μmol m^-2^ s^-1^ and decreased at light intensities higher than 150 μmol m^-2^ s^-1^. Altogether, it is likely that in this work *P*. *tricornutum* becomes photo inhibited at light intensities above 100 μmol m^-2^ s^-1^.

### Changes in TAG accumulation, productivity and yields

Independent of incident light intensity, the TAG concentration increased from 0.05 g L^-1^ (3% of dry weight) under nitrogen replete conditions to ~0.41 g L^-1^ (~25% of dry weight) after prolonged nitrogen starvation (Fig 1 and S2 Table). These maximum TAG contents are in agreement with observations from literature (Table 1).

**Table 1:** Comparison of the maximum TAG and total fatty acid content obtained in this study to literature values. *TAG contents were estimated from the total fatty acid content, assuming 8% of membrane lipids per dry weight.

TAG content	Total fatty acid content	Strain	Reference
(% dry weight)	(% dry weight)		
25	31	*P*. *tricornutum*	This study
22*	30	*P*. *tricornutum*	[4]
20*	28	*P*. *tricornutum*	[26]
26*	34	*P*. *tricornutum*	[27]

Except for the culture exposed to the lowest light intensity (60 μmol m^-2^ s^-1^), TAG accumulation did not differ significantly for the tested conditions (Fig 1). The culture exposed to high incident light intensity (750 μmol m^-2^ s^-1^) showed a decline in TAG content from day 11 onwards, which might indicate photo damage or even cell death.

**Fig 2:**
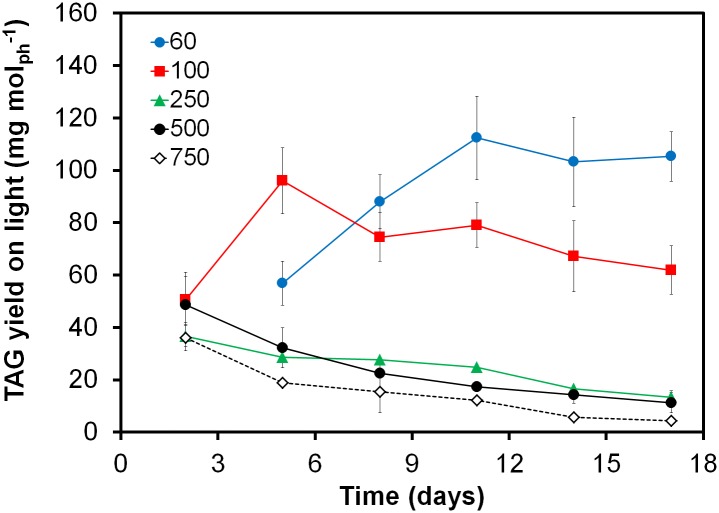
Time averaged TAG yield on light for five different incident light intensities over time. Incident light intensities of 60 μmol m^-2^ s^-1^ (blue circles), 100 μmol m^-2^ s^-1^ (red squares), 250 μmol m^-2^ s^-1^ (green triangles), 500 μmol m^-2^ s^-1^ (black circles) and 750 μmol m^-2^ s^-1^ (open diamonds). Sufficient nitrogen was added to reach biomass concentrations of 1.2 g L^-1^, thereafter cells were exposed to nitrogen starvation. Error bars indicate the standard deviation of both cultures from the average value.

The TAG yield on light is directly proportional to areal productivity and is therefore also strongly related to investment and variable production costs. The maximum time-averaged TAG yield on light is often used as indicator to determine optimal harvest moments [7]. Our study shows that large differences were observed in the maximum time-averaged TAG yield on light among the different tested incident light intensities (Fig 2). Of all tested conditions, the cultures exposed to low light (60 μmol m^-2^ s^-1^) reached the highest maximum TAG yield on light (112 mg TAG mol_ph_^-1^). Increasing the incident light intensity only resulted in lower TAG yields on light: 96 mg TAG mol_ph_^-1^ for the culture exposed to 100 μmol m^-2^ s^-1^ and 36–48 mg TAG mol_ph_^-1^ for cultures exposed to even higher light intensities. In comparison with other studies (Table 2), *P*. *tricornutum* shows relative low TAG yields on light compared to other strains at similar or higher incident light intensities.

**Table 2:** Comparison of the maximum time-averaged TAG yield on light obtained in this study to literature values. CL: continuous light, LD16:8: light dark cycles with 16h of light followed by 8h of darkness. ^1^ was calculated by assuming that the total lipid fraction existed for 90% out of TAG. ^2^ Assumed that 42% (J/J) of the total irradiance has a wavelength in the photosynthetic active radiation spectrum with an energy content of 0.217 MJ mol_ph_^-1^.^3^ Light was supplied with a sinus function with a solar noon at 1500 μmol m^-2^ s^-1^ and a daily averaged light intensity of 636 μmol m^-2^ s^-1^. ^4^ the calculated range is depends on initial biomass concentration at the moment of nitrogen deprivation and includes measurement errors. ^5^ calculation includes the costs for inoculum production (assuming 1 g biomass mol_ph_^-1^).

Species	TAG yield on light	Incident light intensity	Operational strategy	Reference
	(g mol^-1^)	(μmol m^-2^ s^-1^)		
*Phaeodactylum tricornutum*	0.12	60	Batch, LD16:8	This study
*Acutodesmus obliquus*	0.24, 0.13, 0.09, 0.04	200, 500, 800, 1500	Batch, CL	[9]
*Acutodesmus obliquus*	0.15	500	Batch, CL	[39]
*Acutodesmus obliquus slm1*	0.22	500	Batch, CL	[39]
*Acutodesmus obliquus*	0.16	500	Batch, LD16:8	Remmers et al. (submitted)
*Acutodesmus obliquus slm1*	0.20	500	Batch, LD16:8	Remmers et al. (submitted)
*Clorella zofingiensis*	0.32	245	Batch, CL	[5]
*Chlorella vulgaris*	0.16^1^	615^2^	Batch, outdoor	[40]
*Chlorococcum litrorale*	0.16	636^3^	Batch, sinosidial LD 16:8	[41]
*Chlorococcum litrorale*, S5 sorted population	0.32	636^3^	Batch, sinosidial LD 16:8	[41]
*Neochloris oleoabundans*	0.16	218	Batch, CL	[6]
*Nannochloropsis oculata*	0.17–0.25^4^	250	Batch, CL	[42]
*Nannochloropsis sp*.	0.10	636^3^	Batch, sinosidial LD 16:8	[20]
*Nannochloropsis sp*.	0.14^5^	636	Batch, CL	[7]

Sustained biomass productivity upon nitrogen starvation is a key selection parameter for efficient TAG production. As shown in Fig 1A, *P*. *tricornutum* only showed a 2–2.2-fold biomass increase upon nitrogen starvation. Other oleaginous green microalgae (e.g. *Acutodesmus obliquus*, *Chlorella zofingiensis)* showed a 6–8 fold increase in biomass upon nitrogen starvation [4,5,19]. High photon to TAG conversion yields are only possible when light energy is efficiently used to fix carbon (e.g. measured as biomass productivity) and when the majority of fixed carbon is channelled towards storage lipids (e.g. TAG content). Literature reports that diatoms are specifically known to mobilize alternative electron transport (AET) pathways, that can eradicate up to 50% of the electrons released by PS II [28–30]. Also, increased Non-Photochemical Quenching (NPQ) rates were found for *P*. *tricornutum* when exposed to increased light intensities [31–33]. Both processes are known to protect the cells from photo inhibition but to also result in inherent productivity losses. For *P*. *tricornutum*, these mechanisms might explain the reduced photosynthetic efficiency compared to other microalgae under low light nitrogen starvation.

Considering the abovementioned light sensitivity of *P*. *tricornutum* during nitrogen starvation, precautions should be taken to prevent the culture from extensive photo inhibition when working in high irradiance conditions [34]. Examples are to increase the culture cell density to create a dark zone in which the photo inhibitory damage can be repaired [35,36] or to execute strain improvement for reduced antenna size [37,38].

### FA composition

The fatty acid composition of TAG and membrane lipids (ML) did not substantially change over time nor with incident light intensity (the complete fatty acid composition of both fractions during the time-course of the experiments can be found in Table 3). The most abundant fatty acids in TAG were found to be C16:1 (44% of total TAG fatty acids) and C16:0 (30% of total TAG fatty acids). Only a small fraction of C20:5 (EPA) was found in the TAG pool (6–10% of total TAG fatty acids). The membrane lipid fraction was primarily comprised of C16:0 (20% of total ML fatty acids), C16:1 (25–30% of total ML fatty acids) and C20:5 (25–30% of total ML fatty acids) (Table 3). Literature reports similar fatty acid profiles in *P*. *tricornutum* [4,43–45]. As a consequence of TAG accumulation (Fig 1) and the lower EPA content of TAG compared to membrane lipids, the EPA fraction of the total fatty acid pool in the biomass dropped from 25% to 9% of the total fatty acids (S3 Table). Because the biomass productivity declined at the onset of nitrogen depletion, EPA productivity was highest (6–12 mg L^-1^day^-1^) under nitrogen replete growth conditions.

**Table 3:** Average fatty acid composition of *P*. *tricornutum* in the TAG and ML lipid fraction for five different light intensities: 60, 100, 250, 500 and 750 μmol m^-2^ s^-1^. Values are averaged over the entire time course of the experiment (n = 7). Standard deviations are shown between brackets.

	% of fatty acids in TAG fraction					% of fatty acids in ML fraction				
	60	100	250	500	750	60	100	250	500	750
**C14:0**	5.1	5	5.5	5.2	5.8	6.5	5.7	5.5	6.4	6
	(0.7)	(0.5)	(0.9)	(0.5)	(0.4)	(0.3)	(0.7)	(0.8)	(0.4)	(1.2)
**C16:0**	24.8	26.3	31.8	29.8	25	19.1	21.2	25.7	26.3	31.1
	(3.6)	(4.5)	(1.4)	(3.7)	(0.4)	(3.3)	(3.7)	(5.6)	(7.9)	(8.5)
**C16:1**	40.4	44.8	43.1	42	49.1	26.4	26.5	26.6	25.8	21.8
	(5.1)	(3.2)	(3.1)	(5.5)	(3.4)	(2.3)	(2.1)	(2.2)	(1.5)	(4.3)
**C16:2**	0.7	1.3	1.9	0.7	2.1	3	2.5	1.2	1.7	1.2
	(0.5)	(1.3)	(0.4)	(0.3)	(3.1)	(1.1)	(1.9)	(0.6)	(1)	(0.5)
**C16:3**	1.1	1.3	1.2	0.9	1.4	5.2	4.3	3.7	4.1	3.4
	(0.5)	(0.8)	(0.6)	(0.3)	(0.7)	(2.3)	(1.7)	(1.9)	(2.5)	(2.3)
**C18:0**	1.8	1.7	1.6	1.4	1.5	2.4	3.6	5.2	3.4	7.6
	(0.2)	(0.3)	(0.5)	(0.2)	(0.5)	(1)	(1.5)	(2.8)	(1.3)	(5.3)
**C18:1**	5.8	5.7	5.1	4.2	4.6	2.7	3.8	4.1	4	4.5
	(1.2)	(1.4)	(1.1)	(0.6)	(1.1)	(1.1)	(2.4)	(1.7)	(1.7)	(1.6)
**C18:2w6**	1.4	1.3	1.3	1.1	1.3	2	1.4	2.2	2	2
	(0.3)	(0.3)	(0.2)	(0.3)	(0.2)	(0.6)	(0.4)	(0.8)	(0.8)	(1.1)
**C18:3w3**	6.7	3.3	2.8	5.6	2.7	1.5	1.5	1.7	1.3	1.5
	(4.3)	(2.4)	(0.6)	(5.4)	(0.6)	(0.4)	(0.6)	(0.5)	(0.3)	(0.8)
**C20:3'(n3**	0.9	0.4	0.1	1.7	0.4	5.8	5.7	3.8	3.7	3
	(0.8)	(0.3)	(0.1)	(4)	(0.8)	(1.7)	(2.7)	(1.3)	(0.7)	(2.1)
**C20:5'(n3**	7.8	7.1	6.7	7.4	7.2	25.5	23.8	20.3	21.3	18
	(1.4)	(1.4)	(0.8)	(1)	(1.1)	(5.7)	(7)	(5.5)	(7.1)	(8.9)

### EPA incorporation in membrane lipids and triacylglycerol

**Fig 3:**
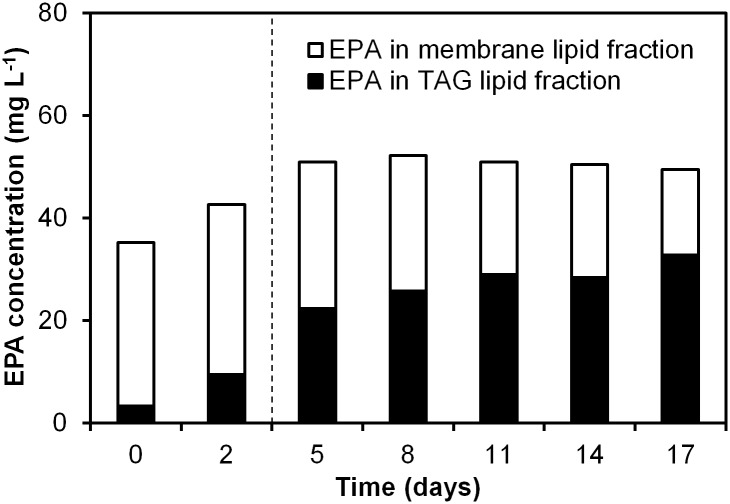
EPA concentration in both TAG and ML lipids over time at an incident light intensity of 100 μmol m^-2^ s^-1^. The moment of nitrogen starvation is indicated with a dashed line.

Fig 3 shows the EPA concentration per litre culture with respect to the TAG en membrane lipid fraction. Apart from a small increase until day 5, neither a substantial net synthesis nor breakdown of EPA upon N starvation is observed. However, the distribution of EPA over TAG and membrane lipids changes over time. Upon nitrogen depletion, a substantial increase in EPA distribution towards TAG can be observed. Simultaneously, we observed a decrease in EPA that was bound to membrane lipids. Therefore, we hypothesize that the EPA found in TAG under nitrogen starvation could partially be derived from EPA-containing membrane lipids that were restructured upon nitrogen depletion. Similar observations have been made for *Nannochloropsis oceanica* [46], *Nannochloropsis oculata* and *Pavlova lutheri* [47].

For many species, nitrogen depletion results in an inherent degradation of photosynthetic membrane lipids with simultaneous accumulation of TAG. Part of the fatty acids originating from membrane lipids could thus be used as fatty acyl donors for TAG synthesis under nutrient deprivation (Fig 3). However, detailed knowledge on the mechanisms behind carbon partitioning in microalgae under nitrogen deprivation is still lacking [47–51] and further research using ^13^C labelling should confirm these observations. To further improve EPA productivity by means of metabolic engineering or process development [52], it is crucial to understand the regulation of EPA towards different lipids classes (e.g. TAG or structural membrane lipids) under different environmental conditions.

## Conclusion

Elevated light intensities (>100 μmol m^-2^ s^-1^) reduce the photosynthetic efficiency and the TAG yield on light during nitrogen starvation, likely caused by photo inhibition. Compared to other microalgae strains, relative low TAG yields on light were observed for *P*. *tricornutum* as the maximum yield was only 112 mg TAG mol_ph_^-1^ (at 60 μmol m^-2^ s^-1^). TAG contents were similar for all light intensities tested (i.e. 25% of dry weight). Further analysis on the fatty acid composition revealed that the TAG fraction of *P*. *tricornutum* contains 6–10% of EPA. Mass balance analysis revealed this EPA fraction may originate partly from photosynthetic membrane lipids that were restructured after nitrogen depletion.

## Supporting information

S1 Fig Emission spectrum of the Algaemist Bridgelux LED panel. Data obtained from de Mooij et al (2014).
(TIF)

S1 Table Carbohydrate content over time for five different incident light intensities. For all experiments n = 2, except for incident light intensity of 250 and 750 μmol m^-2^ s^-1^ with n = 1. Values in brackets represent the standard deviation from the two biological duplicates.
(DOCX)

S2 Table TAG concentration over time for five different incident light intensities. For all experiments n = 2, except for incident light intensity of 250 and 750 μmol m-2 s-1 with n = 1. Values in brackets represent the standard deviation from the two biological duplicates.
(DOCX)

S3 Table EPA content in the total lipid fraction over time for five different incident light intensities. For all experiments n = 2, except for incident light intensity of 250 and 750 μmol m-2 s-1 with n = 1. Values in brackets represent the standard deviation from the two biological duplicates.
(DOCX)

## References

[1] Ruiz J, Olivieri G, de Vree J, Bosma R, Willems P, Reith JH, et al Towards industrial products from microalgae. Energy Environ Sci. 2016;9: 3036–3043.

[2] Chauton MS, Reitan KI, Norsker NH, Tveterås R, Kleivdal HT. A techno-economic analysis of industrial production of marine microalgae as a source of EPA and DHA-rich raw material for aquafeed: Research challenges and possibilities. Aquaculture. 2015;436: 95–103.

[3] Norsker N-H, Barbosa MJ, Vermuë MH, Wijffels RH. Microalgal production—A close look at the economics. Biotechnol Adv. 2011;29: 24–27. 10.1016/j.biotechadv.2010.08.005 20728528

[4] Breuer G, Lamers PP, Martens DE, Draaisma RB, Wijffels RH. The impact of nitrogen starvation on the dynamics of triacylglycerol accumulation in nine microalgae strains. Bioresour Technol. 2012;124: 217–26. 10.1016/j.biortech.2012.08.003 22995162

[5] Mulders KJM, Janssen JH, Martens DE, Wijffels RH, Lamers PP. Effect of biomass concentration on secondary carotenoids and triacylglycerol (TAG) accumulation in nitrogen-depleted Chlorella zofingiensis. Algal Res. 2014;6: 8–16.

[6] Santos AM, Wijffels RH, Lamers PP. pH-upshock yields more lipids in nitrogen-starved Neochloris oleoabundans. Bioresour Technol. 2014;152: 299–306. 10.1016/j.biortech.2013.10.079 24296123

[7] Benvenuti G, Lamers PP, Breuer G, Bosma R, Cerar A, Wijffels RH, et al Microalgal TAG production strategies: why batch beats repeated-batch. Biotechnol Biofuels. 2016;9: 64 10.1186/s13068-016-0475-4 26985237 PMC4793540

[8] Bitaubé Pérez E, Caro Pina I, Pérez Rodríquez L. Kinetic model for growth of Phaeodactylum tricornutum in intensive culture photobioreactor. Biochem Eng J. 2008;40: 520–525.

[9] Breuer G, Lamers PP, Martens DE, Draaisma RB, Wijffels RH. Effect of light intensity, pH, and temperature on triacylglycerol (TAG) accumulation induced by nitrogen starvation in Scenedesmus obliquus. Bioresour Technol. 2013;143: 1–9. 10.1016/j.biortech.2013.05.105 23774290

[10] Molina-Grima E, Pérez JAS, Camacho FG, Fernández FGA, Sevilla JMF, Sanz FV. Effect of dilution rate on eicosapentaenoic acid productivity of Phaeodactylum tricornutum utex 640 in outdoor chemostat culture. Biotechnol Appl Biochem. Kluwer Academic Publishers; 1994;16: 1035–1040.

[11] Reis A, Gouveia L, Veloso V, Fernandes HL, Empis JA, Novais JM. Eicosapentaenoic acid-rich biomass production by the microalga Phaeodactylum tricornutum in a continuous-flow reactor. Bioresour Technol. 1996;55: 83–88.

[12] Yongmanitchal W, Ward OP. Growth and eicosapentaenoic acid production by Phaeodactylum tricornutum in batch and continuous culture systems. J Am Oil Chem Soc. Springer-Verlag; 1992;69: 584–590.

[13] Geider J. R, Osborne BA, Raven JA. Light Dependence of Growth and Photosynthesis in Phaeodactylum Tricornutum (bacillariophyceae). J Phycol. 1985;21: 609–619.

[14] Ritchie RJ. Fitting light saturation curves measured using modulated fluorometry. Photosynth Res. 2008;96: 201–215. 10.1007/s11120-008-9300-7 18415696

[15] Wiegman S, Barranguet C, Spijkerman E, Kraak MHS, Admiraal W. The role of ultraviolet-adaptation of a marine diatom in photoenhanced toxicity of acridine. Environ Toxicol Chem. 2003;22: 591–598. 12627647

[16] Postma PR, Miron TL, Olivieri G, Barbosa MJ, Wijffels RH, Eppink MHM. Mild disintegration of the green microalgae Chlorella vulgaris using bead milling. Bioresour Technol. 2015;184: 297–304. 10.1016/j.biortech.2014.09.033 25280602

[17] Lamers PP, van de Laak CCW, Kaasenbrood PS, Lorier J, Janssen M, De Vos RCH, et al Carotenoid and fatty acid metabolism in light-stressed Dunaliella salina. Biotechnol Bioeng. 2010;106: 638–48. 10.1002/bit.22725 20229508

[18] de Mooij T, Janssen M, Cerezo-Chinarro O, Mussgnug JH, Kruse O, Ballottari M, et al Antenna size reduction as a strategy to increase biomass productivity: a great potential not yet realized. J Appl Phycol. 2014;27: 1063–1077.

[19] Benvenuti G, Bosma R, Cuaresma M, Janssen M, Barbosa MJ, Wijffels RH. Selecting microalgae with high lipid productivity and photosynthetic activity under nitrogen starvation. J Appl Phycol. 2014;27: 1425–1431.

[20] Benvenuti G, Bosma R, Ji F, Lamers P, Barbosa MJ, Wijffels RH. Batch and semi-continuous microalgal TAG production in lab-scale and outdoor photobioreactors. J Appl Phycol. 2016;28: 3167–3177. 10.1007/s10811-016-0897-1 28035172 PMC5155026

[21] Breuer G, Lamers PP, Janssen M, Wijffels RH, Martens DE. Opportunities to improve the areal oil productivity of microalgae. Bioresour Technol. 2015;186: 294–302. 10.1016/j.biortech.2015.03.085 25836038

[22] Dillschneider R, Steinweg C, Rosello-Sastre R, Posten C. Biofuels from microalgae: Photoconversion efficiency during lipid accumulation. Bioresour Technol. 2013;142: 647–654. 10.1016/j.biortech.2013.05.088 23777817

[23] Parkhill J-P, Maillet G, Cullen JJ. Fluorescence-based maximal quantum yield for PSII as a diagnostic of nutrient stress. J Phycol. 2001;37: 517–529.

[24] Zhao Y, Wang Y, Quigg A. Comparison of population growth and photosynthetic apparatus changes in response to different nutrient status in a diatom and a coccolithophore. Wetherbee R, editor. J Phycol. 2015;51: 872–884. 10.1111/jpy.12327 26986884

[25] Acién Fernández FG, Hall DO, Cañizares Guerrero E, Krishna Rao K, Molina Grima E. Outdoor production of Phaeodactylum tricornutum biomass in a helical reactor. J Biotechnol. 2003;103: 137–152. 10.1016/S0168-1656(03)00101-9 12814873

[26] Griffiths MJ, van Hille RP, Harrison STL. Lipid productivity, settling potential and fatty acid profile of 11 microalgal species grown under nitrogen replete and limited conditions. J Appl Phycol. 2011;24: 989–1001.

[27] Santos-Ballardo DU, Rendón-Unceta MDC, Rossi S, Vázquez-Gómez R, Hernández-Verdugo S, Valdez-Ortiz A. Effects of outdoor cultures on the growth and lipid production of Phaeodactylum tricornutum using closed photobioreactors. World J Microbiol Biotechnol. 2016;32: 128 10.1007/s11274-016-2089-1 27339309

[28] Jallet D, Caballero MA, Gallina AA, Youngblood M, Peers G. Photosynthetic physiology and biomass partitioning in the model diatom Phaeodactylum tricornutum grown in a sinusoidal light regime. Algal Res. 2016;18: 51–60.

[29] Wagner H, Jakob T, Lavaud J, Wilhelm C. Photosystem II cycle activity and alternative electron transport in the diatom Phaeodactylum tricornutum under dynamic light conditions and nitrogen limitation. Photosynth Res. 2015;128: 151–161. 10.1007/s11120-015-0209-7 26650230

[30] Waring J, Klenell M, Bechtold U, Underwood GJC, Baker NR. Light-Induced Responses of Oxygen Photoreduction, Reactive Oxygen Species Production and Scavenging in Two Diatom Species. J Phycol. 2010;46: 1206–1217.

[31] Bailleul B, Rogato A, de Martino A, Coesel S, Cardol P, Bowler C, et al An atypical member of the light-harvesting complex stress-related protein family modulates diatom responses to light. Proc Natl Acad Sci. 2010;107: 18214–18219. 10.1073/pnas.1007703107 20921421 PMC2964204

[32] Chauton MS, Winge P, Brembu T, Vadstein O, Bones AM. Gene regulation of carbon fixation, storage, and utilization in the diatom Phaeodactylum tricornutum acclimated to light/dark cycles. Plant Physiol. 2013;161: 1034–48. 10.1104/pp.112.206177 23209127 PMC3561001

[33] Lavaud J, Rousseau B, van Gorkom HJ, Etienne A-L. Influence of the Diadinoxanthin Pool Size on Photoprotection in the Marine Planktonic Diatom Phaeodactylum tricornutum. Plant Physiol. 2002;129: 1398–1406. 10.1104/pp.002014 12114593 PMC166533

[34] Richmond A. Handbook of Microalgal Culture: Biotechnology and Applied Phycology. John Wiley & Sons; 2008.

[35] Olivieri G, Salatino P, Marzocchella A. Advances in photobioreactors for intensive microalgal production: configurations, operating strategies and applications. J Chem Technol Biotechnol. 2014;89: 178–195.

[36] Tredici MR, Zittelli GC. Efficiency of sunlight utilization: Tubular versus flat photobioreactors. Biotechnol Bioeng. 1998;57: 187–197. 10099193 10.1002/(sici)1097-0290(19980120)57:2<187::aid-bit7>3.0.co;2-j

[37] de Mooij T, Schediwy K, Wijffels RH, Janssen M. Modeling the competition between antenna size mutant and wild type microalgae in outdoor mass culture. J Biotechnol. 2016;240: 1–13. 10.1016/j.jbiotec.2016.10.009 27746308

[38] Perin G, Bellan A, Segalla A, Meneghesso A, Alboresi A, Morosinotto T. Generation of random mutants to improve light-use efficiency of Nannochloropsis gaditana cultures for biofuel production. Biotechnol Biofuels. 2015;8. 10.1186/s13068-015-0337-5 PMC4583171 26413160

[39] Breuer G, de Jaeger L, Artus VPG, Martens DE, Springer J, Draaisma RB, et al Superior triacylglycerol (TAG) accumulation in starchless mutants of Scenedesmus obliquus: (II) evaluation of TAG yield and productivity in controlled photobioreactors. Biotechnol Biofuels. 2014;7: 70 10.1186/1754-6834-7-70 24883102 PMC4026393

[40] Münkel R, Schmid-Staiger U, Werner A, Hirth T. Optimization of outdoor cultivation in flat panel airlift reactors for lipid production by Chlorella vulgaris. Biotechnol Bioeng. 2013;110: 2882–2893. 10.1002/bit.24948 23616347

[41] Cabanelas ITD, van der Zwart M, Kleinegris DMM, Wijffels RH, Barbosa MJ. Sorting cells of the microalga Chlorococcum littorale with increased triacylglycerol productivity. Biotechnol Biofuels. 2016;9: 183 10.1186/s13068-016-0595-x 27582875 PMC5006580

[42] Van Vooren G, Le Grand F, Legrand J, Cuiné S, Peltier G, Pruvost J. Investigation of fatty acids accumulation in Nannochloropsis oculata for biodiesel application. Bioresour Technol. 2012;124: 421–432. 10.1016/j.biortech.2012.08.009 23018107

[43] Abida H, Dolch L-J, Meï C, Villanova V, Conte M, Block MA, et al Membrane Glycerolipid Remodeling Triggered by Nitrogen and Phosphorus Starvation in Phaeodactylum tricornutum. Plant Physiol. 2015;167: 118–136. 10.1104/pp.114.252395 25489020 PMC4281014

[44] Alonso DL, Belarbi E-H, Fernández-Sevilla JM, Rodríguez-Ruiz J, Grima EM. Acyl lipid composition variation related to culture age and nitrogen concentration in continuous culture of the microalga Phaeodactylum tricornutum. Phytochemistry. 2000;54: 461–471. 10.1016/S0031-9422(00)00084-4 10939349

[45] Gatenby CM, Orcutt DM, Kreeger DA, Parker BC, Jones VA, Neves RJ. Biochemical composition of three algal species proposed as food for captive freshwater mussels. J Appl Phycol. 2003;15: 1–11.

[46] Jia J, Han D, Gerken HG, Li Y, Sommerfeld M, Hu Q, et al Molecular mechanisms for photosynthetic carbon partitioning into storage neutral lipids in Nannochloropsis oceanica under nitrogen-depletion conditions. Algal Res. 2015;7: 66–77.

[47] Guihéneuf F, Stengel DB. LC-PUFA-enriched oil production by microalgae: accumulation of lipid and triacylglycerols containing n-3 LC-PUFA is triggered by nitrogen limitation and inorganic carbon availability in the marine haptophyte Pavlova lutheri. Mar Drugs. 2013;11: 4246–66. 10.3390/md11114246 24177672 PMC3853726

[48] Juergens MT, Disbrow B, Shachar-Hill Y. The Relationship of Triacylglycerol and Starch Accumulation to Carbon and Energy Flows during Nutrient Deprivation in Chlamydomonas reinhardtii. Plant Physiol. 2016;171: 2445–2457. 10.1104/pp.16.00761 27325664 PMC4972295

[49] Klein U. Intracellular Carbon Partitioning in Chlamydomonas reinhardtii. Plant Physiol. 1987;85: 892–897. 16665826 10.1104/pp.85.4.892 PMC1054364

[50] Mühlroth A, Li K, Røkke G, Winge P, Olsen Y, Hohmann-Marriott MF, et al Pathways of lipid metabolism in marine algae, co-expression network, bottlenecks and candidate genes for enhanced production of EPA and DHA in species of Chromista. Mar Drugs. 2013;11: 4662–97. 10.3390/md11114662 24284429 PMC3853752

[51] Tonon T, Harvey D, Larson TR, Graham IA. Long chain polyunsaturated fatty acid production and partitioning to triacylglycerols in four microalgae. Phytochemistry. 2002;61: 15–24. 12165297 10.1016/s0031-9422(02)00201-7

[52] Adarme-Vega TC, Lim DKY, Timmins M, Vernen F, Li Y, Schenk PM. Microalgal biofactories: a promising approach towards sustainable omega-3 fatty acid production. Microb Cell Factories. 2012;11: 96. 10.1186/1475-2859-11-96 PMC3465194 22830315

