# Supplementary figures and images for: Dynamics of triacylglycerol and EPA production in Phaeodactylum tricornutum under nitrogen starvation at different light intensities
Source: PLoS One. 2017 Apr 12;12(4):e0175630. doi: 10.1371/journal.pone.0175630 (PMC5389818; doi:10.1371/journal.pone.0175630)

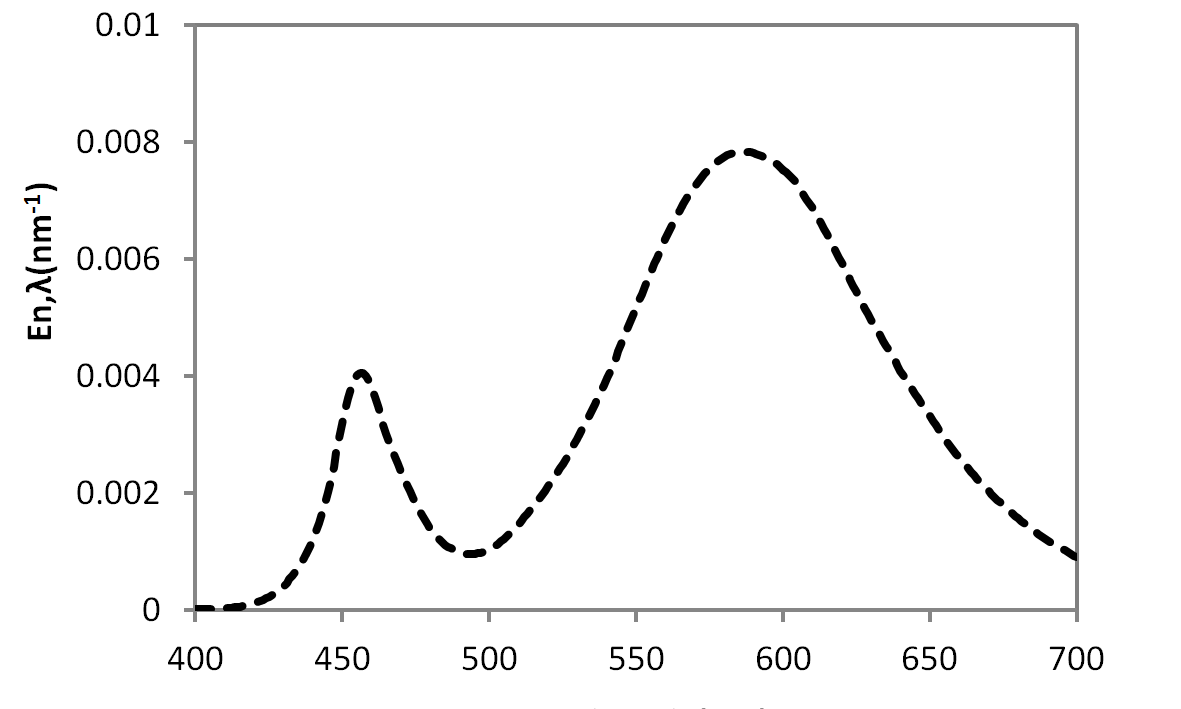

Supplement: S1 Fig — Data obtained from de Mooij et al (2014). (TIF) [file pone.0175630.s001.tif]
