# Supplementary material for: Dynamics of triacylglycerol and EPA production in Phaeodactylum tricornutum under nitrogen starvation at different light intensities
Source: PLoS One. 2017 Apr 12;12(4):e0175630. doi: 10.1371/journal.pone.0175630 (PMC5389818; doi:10.1371/journal.pone.0175630)
Supplement: S1 Table — For all experiments n = 2, except for incident light intensity of 250 and 750 μmol m-2 s-1 with n = 1. Values in brackets represent the standard deviation from the two biological duplicates. (DOCX) [file pone.0175630.s002.docx]

| **Days** | **Total carbohydrate content (%w/w)** | | | | |
| --- | --- | --- | --- | --- | --- |
|  | **60** | **100** | **250** | **500** | **750** |
|  | **µmol m^-2^ s^-1^** | **µmol m^-2^ s^-1^** | **µmol m^-2^ s^-1^** | **µmol m^-2^ s^-1^** | **µmol m^-2^ s^-1^** |
| **0** | 15.23  (1.18) | 12.10  (1.09) | 12.9 | 12.53  (0.70) | 15.00 |
| **2** | 18.18  (0.59) | 20.64  (2.45) | 21.83 | 25.64  (6.64) | 23.2 |
| **5** | 28.61  (1.99) | 24.17  (6.19) | 20.08 | 25.78  (10.41) | 18.53 |
| **8** | 32.21  (0.62) | 28.22  (4) | 23.97 | 26.92  (6.26) | 26.42 |
| **11** | 33.01  (1.46) | 26.96  (4.22) | 23.31 | 25.29  (5.11) | 25.10 |
| **15** | 27.48  (0.80) | 22.98  (2.44) | 22.76 | 23.85  (1.94) | 27.56 |
| **17** | 28.82  (0.89) | 19.37  (2.66) | 19.37 | 21.69  (5.56) | 26.11 |
