# Supplementary material for: Dynamics of triacylglycerol and EPA production in Phaeodactylum tricornutum under nitrogen starvation at different light intensities
Source: PLoS One. 2017 Apr 12;12(4):e0175630. doi: 10.1371/journal.pone.0175630 (PMC5389818; doi:10.1371/journal.pone.0175630)
Supplement: S2 Table — For all experiments n = 2, except for incident light intensity of 250 and 750 μmol m-2 s-1 with n = 1. Values in brackets represent the standard deviation from the two biological duplicates. (DOCX) [file pone.0175630.s003.docx]

| **Days** | **TAG concentration (g L^-1^)** | | | | |
| --- | --- | --- | --- | --- | --- |
|  | **60**  **µmol m^-2^ s^-1^** | **100**  **µmol m^-2^ s^-1^** | **250**  **µmol m^-2^ s^-1^** | **500**  **µmol m^-2^ s^-1^** | **750**  **µmol m^-2^ s^-1^** |
| **0** | 0.04  (0.01) | 0.03  (0) | 0.05 | 0.05  (0.01) | 0.04 |
| **2** | 0.04  (0) | 0.13  (0.09) | 0.16 | 0.26  (0.07) | 0.21 |
| **5** | 0.14  (0.03) | 0.33  (0.05) | 0.23 | 0.39  (0.11) | 0.30 |
| **8** | 0.26  (0.03) | 0.36  (0.04) | 0.31 | 0.43  (0.06) | 0.39 |
| **11** | 0.4  (0.06) | 0.48  (0.05) | 0.36 | 0.44  (0.02) | 0.43 |
| **14** | 0.45  (0.07) | 0.49  (0.11) | 0.31 | 0.46  (0.06) | 0.27 |
| **17** | 0.54  (0.07) | 0.53  (0.16) | 0.30 | 0.45  (0.1) | 0.26 |
