# Supplementary material for: Dynamics of triacylglycerol and EPA production in Phaeodactylum tricornutum under nitrogen starvation at different light intensities
Source: PLoS One. 2017 Apr 12;12(4):e0175630. doi: 10.1371/journal.pone.0175630 (PMC5389818; doi:10.1371/journal.pone.0175630)
Supplement: S3 Table — For all experiments n = 2, except for incident light intensity of 250 and 750 μmol m-2 s-1 with n = 1. Values in brackets represent the standard deviation from the two biological duplicates. (DOCX) [file pone.0175630.s004.docx]

| **Days** | **EPA content in total fatty acids (%w/w)** | | | | |
| --- | --- | --- | --- | --- | --- |
|  | **60** | **100** | **250** | **500** | **750** |
|  | **µmol m^-2^ s^-1^** | **µmol m^-2^ s^-1^** | **µmol m^-2^ s^-1^** | **µmol m^-2^ s^-1^** | **µmol m^-2^ s^-1^** |
| **0** | 24.97  (0) | 18.86  (8.64) | 12.75 | 21.81  (4.47) | 12.75 |
| **2** | 23.08  (4) | 22.13  (10.53) | 15.71 | 13.36  (0) | 22.07 |
| **5** | 23.43  (3.48) | 17.47  (4.67) | 13.92 | 12.93  (0.86) | 10.79 |
| **8** | 16.42  (0.4) | 10.44  (3.64) | 11.50 | 12.15  (1.02) | 12.21 |
| **11** | 14.96  (0.76) | 11.04  (1.42) | 11.25 | 11.88  (0.4) | 12.16 |
| **15** | 11.21  (0.35) | 9.09  (2.06) | 9.90 | 9.66  (0.89) | 8.06 |
| **17** | 9.11  (0.26) | 8.23  (2.17) | 8.72 | 8.77  (0.72) | 7.89 |
